# Cancer risks from chest radiography of young adults: A pilot study at a health facility in South West Nigeria

**DOI:** 10.1016/j.dib.2018.05.123

**Published:** 2018-05-26

**Authors:** Justina A. Achuka, Moses A. Aweda, Mojisola R. Usikalu, Caleb A. Aborisade

**Affiliations:** a Department of Physics, Covenant University, Ota, Ogun State, Nigeria; b Department of Radiation Biology, Radiotherapy and Radiodiagnosis, College of Medicine, Lagos University Teaching Hospital, Idi-Araba, Lagos, Nigeria; c Department of Physics and Engineering Physics, Obafemi Awolowo University, Ile-Ife, Osun State, Nigeria

**Keywords:** Chest radiography, Entrance surface dose, Incidence cancer risks, Technical factors

## Abstract

The recommendation of chest radiography for school admission and employment purposes should be discouraged due to the risks of radiation especially cancer induction. It is therefore imperative to keep diagnostic radiation doses as low as possible. This dataset presents the entrance surface dose, effective dose, bone marrow dose, breast dose, lung dose and the incidence cancer risks from chest radiography of 40 young adult females. The mean incidence cancer risk to participants is 1: 20,000 for solid cancers. The data revealed the significant factors influencing the entrance surface dose and incidence cancer risks.

## Specifications Table

**Table:** 

Subject area	Medicine
More specific subject area	Diagnostic Radiology, X-ray Imaging, Radiation dosimetry, Radiation Protection
Type of data	Tables and figures
How data was acquired	Thermoluminescent dosimeters (TLD-100; RadPro, Poland), PCXMC Software (20Rotation), Quality Control Kits (MagicMax, Germany)
Data format	Raw, Analyzed
Experimental factors	The aforementioned parameters in the abstracts were analyzed according to International Atomic Energy Agency (IAEA) standards for radiation protection of patients
Experimental features	Determination of entrance surface dose, effective dose and bone marrow dose, breast dose and lung dose in order to estimate the risk of radiation induced cancer from chest radiography
Data source location	Obafemi Awolowo University Teaching Hospital Complex, Ile-Ife, Osun State, Nigeria
Data accessibility	All the data are in this data article

## Value of the data

• The data can be used to assess incidence cancer risk from chest radiography in the State.
• The data will help to curtail the demand for chest radiography for school admission and employment purposes.
• The data will enhance the optimization of radiographic procedures in the State to be as low as reasonably achievable.
• The data is useful in radiation protection training and epidemiology studies.
• Cancer risks assessment can be extended to other irradiated organs arising from chest radiography not covered in this study.
• The study can be extended to multi-centre studies.
• The data can be helpful to radiation regulatory authorities and policy makers.

## 1 Data

The data contains radiation doses and incidence of cancer risks among young adult females who underwent chest radiography for school admission purposes. Radiation protection of patients in diagnostic radiology is a subject of global concern. Concerted effort to minimizing patient׳s dose has led to generation of datasets [1], [2], [3], [4], [5]. Justification of radiographic examinations and optimization of the procedures have been the emphasis for the protection of patients [2], [5], [6]. Data on some experiences leading to the discouragement of requests for chest radiography used for school admission and employment purposes can be found in [7], [8], [9]. Data on the risks of cancer induction from low dose ionizing radiation can be found in [10], [11], [12], [13], [14]. Beyond cancer induction other radiation risks have been reported [15], [16], [17].

### 1.1 Description of data

**Fig. 1:**
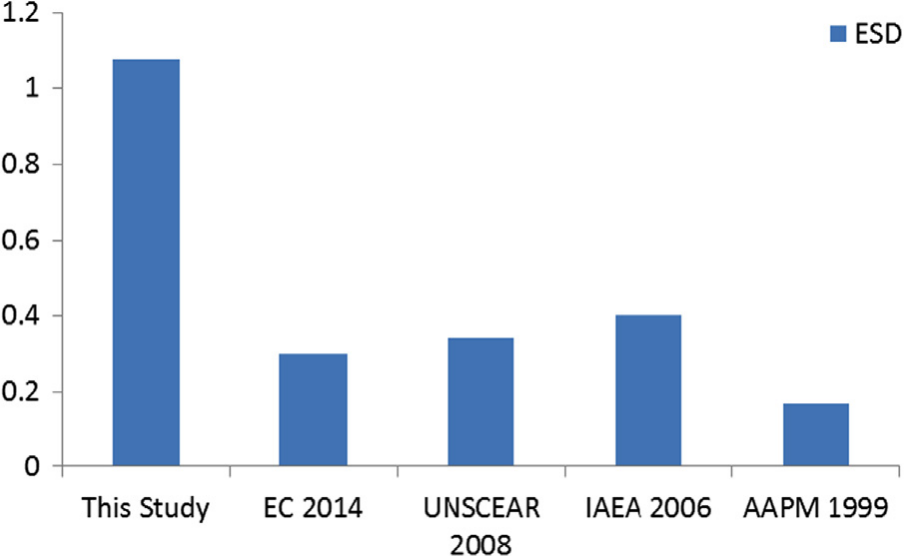
Comparison of entrance surface dose [3], [18], [19], [20].

**Fig. 2:**
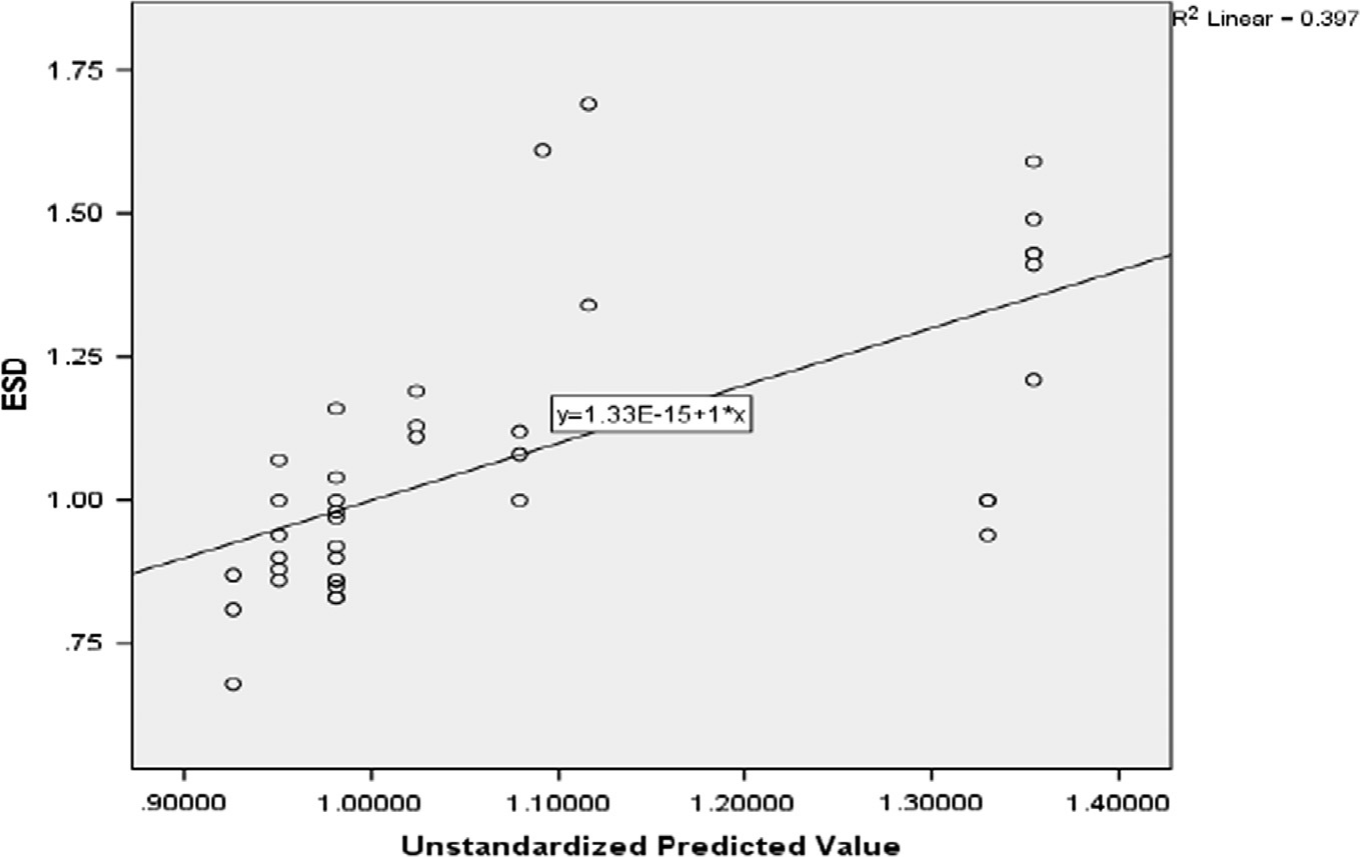
Scatter line plot for entrance surface dose (ESD), focus to film distance (FFD) and current time product (mAs).

The patient parameters, technical factors, radiation doses and incidence cancer risks are presented in Tables 1, Table 1, Table 6. Descriptive analysis of patient parameters and technical factors are presented in Table 2 and the descriptive analysis of radiation doses and cancer risks is reported in Table 7. The influence of patient parameters and technical factors on entrance surface dose (ESD) is reported in Table 2, Table 3, Table 4, Table 5 and Fig. 2. Fig. 1 compares the entrance surface dose (ESD) with world data (Table 7). The cancer risks ratio is presented in Table 7.

**Table 1:** Descriptive statistics of patient parameters and technical factors.

		**Age**	**BMI**	**FFD**	**FSD**	**kVp**	**mAs**
*N*	Valid	40	40	40	40	40	40
	Missing	0	0	0	0	0	0
Mean		20.25	22.8815	147.23	121.58	74.13	23.50
Median		20.00	22.2700	153.00	124.00	74.00	25.00
Std. Deviation		2.295	3.82241	8.710	7.828	2.633	4.591
Variance		5.269	14.611	75.871	61.276	6.933	21.077
Skewness		0.360	0.321	− 1.143	− 0.267	0.115	0.510
Std. Error of Skewness		0.374	0.374	0.374	0.374	0.374	0.374
Kurtosis		− 1.016	− 0.622	− 0.467	− 1.311	− 0.794	− 0.341
Std. Error of Kurtosis		0.733	0.733	0.733	0.733	0.733	0.733
Range		8	14.84	22	26	9	16
Minimum		17	15.63	131	109	70	16
Maximum		25	30.47	153	135	79	32

ESD = entrance surface dose; BD = breast dose; ICR = incidence cancer risks; FFD = focus film distance; FSD = focus skin distance; kVp = kilovoltage peak; mAs = current time product.

**Table 2:** Model Summary for entrance surface dose, patient parameters and technical factors.

Model	*R*	*R* square	Adjusted *R* square	Std. error of the estimate
	0.775	0.601	0.528	0.16941

**Table 3:** Analysis of variance for entrance surface dose, patient parameters and technical factors.

Model		Sum of squares	d*f*	Mean square	*F*	Sig.
1	Regression	1.425	6	0.237	8.275	0.000
	Residual	0.947	33	0.029		
	Total	2.372	39

**Table 4:** Coefficients of variables.

Model		Unstandardized coefficients		Standardized coefficients	*t*	Sig.
		*B*	Std. error	Beta		
1	(Constant)	1.739	2.033		0.855	0.399
	Age	0.024	0.020	0.225	1.225	0.229
	BMI	0.024	0.017	0.371	1.427	0.163
	FFD	− 0.019	0.006	− 0.666	− 3.284	0.002
	FSD	0.013	0.007	0.428	1.908	0.065
	kVp	0.002	0.033	0.022	0.063	0.950
	mAs	− 0.030	0.012	− 0.563	− 2.565	0.015

**Table 5:** Correlation matrix of entrance surface dose, patient parameters and technical factors.

**Correlations**		**ESD**	**Age**	**BMI**	**FFD**	**FSD**	**kVp**	**mAs**
**Pearson**	**ESD**	1						
	**Age**	0.539	1					
	**BMI**	0.234	0.568	1				
	**FFD**	− 0.620	− 0.380	0.039	1			
	**FSD**	− 0.129	0.163	0.442	0.607	1		
	**kVp**	0.379	0.741	0.859	− 0.129	0.502	1	
	**mAs**	− 0.046	0.418	0.793	0.247	0.646	0.760	1
**Kendall׳s**	**ESD**	1						
	**Age**	0.450	1					
	**BMI**	0.221	0.355	1				
	**FFD**	− 0.544	− 0.384	− 0.095	1			
	**FSD**	− 0.011	0.190	0.270	0.409	1		
	**kVp**	0.368	0.596	0.641	− 0.263	0.330	1	
	**mAs**	0.037	0.301	0.627	0.154	0.545	0.587	1
**Spearman׳s**	**ESD**	1						
	**Age**	0.620	1					
	**BMI**	0.314	0.491	1				
	**FFD**	− 0.685	− 0.479	− 0.098	1			
	**FSD**	− 0.050	0.242	0.395	0.482	1		
	**kVp**	0.506	0.753	0.777	− 0.302	0.445	1	
	**mAs**	0.028	0.375	0.734	0.175	0.687	0.674	1

ESD = entrance surface dose; BD = breast dose; ICR = incidence cancer risks; FFD = focus film distance; FSD = focus skin distance; kVp = kilovoltage peak; mAs = current time product.

**Table 6:** Descriptive statistics of radiation doses and cancer risks incidence.

			**ESD**	**E**	**BMD**	**BD**	**LD**	**ICR**_**BM**_	**ICR**_**B**_	**ICR**_**L**_	**ICR**_**S**_
*N*	Valid		40	40	40	40	40	40	40	40	40
	Missing		0	0	0	0	0	0	0	0	0
Mean			1.08	0.16	0.18	0.19	0.66	0.78	0.81	2.81	4.56
Median			1.00	0.15	0.17	0.18	0.61	0.73	0.81	2.82	4.35
Std. Deviation			0.247	0.043	0.043	0.067	0.184	0.162	0.299	0.799	0.879
Variance			0.061	0.002	0.002	0.004	0.034	0.026	0.089	0.638	0.772
Skewness			0.959	1.628	1.334	1.174	1.558	1.559	0.799	1.169	1.255
Std. Error of Skewness			0.374	0.374	0.374	0.374	0.374	0.374	0.374	0.374	0.374
Kurtosis			0.154	2.773	1.058	1.942	2.615	2.601	1.346	2.339	1.405
Std. Error of Kurtosis			0.733	0.733	0.733	0.733	0.733	0.733	0.733	0.733	0.733
Range			1.01	0.20	0.18	0.32	0.86	0.75	1.40	3.92	4.01
Minimum			0.68	0.11	0.13	0.09	0.43	0.58	0.35	1.61	3.24
Maximum			1.69	0.31	0.31	0.41	1.29	1.34	1.75	5.53	7.25
Percentiles		25	0.89	0.14	0.16	0.14	0.54	0.67	0.54	2.17	3.94
		50	1.00	0.15	0.17	0.18	0.61	0.72	0.81	2.82	4.35
		75	1.18	0.17	0.20	0.22	0.70	0.85	0.97	3.26	4.93

ESD = entrance surface dose; E = effective dose; BMD = bone marrow dose; BD = breast dose; LD = lung dose; ICR_BM_ = incidence cancer risks for bone marrow; ICR_B_ = incidence cancer risks for breast; ICR_L_ = incidence cancer risks for lung; ICR_s_ = incidence cancer risks for solid cancers.

**Table 7:** Incidence cancer risks ratio for chest radiography.

		**ICR**_**BM**_	**Ratio**	**ICR**_**B**_	**Ratio**	**ICR**_**L**_	**Ratio**	**ICR**_**S**_	**Ratio**
Mean		0.78	1:100000	0.81	1:100000	2.81	3:100000	4.56	5:100000
Minimum		0.58		0.35		1.61	2:100000	3.24	3:100000
Maximum		1.34	1:100000	1.75	2:100000	5.53	6:100000	7.25	7:100000
Percentiles	25	0.67		0.54		2.17	2:100000	3.94	4:100000
	50	0.73		0.81	1:100000	2.82	3:100000	4.35	4:100000
	75	0.85	1:100000	0.97	1:100000	3.26	3:100000	4.93	5:100000
***Level of Risk:***									
1: 1,000,000–1: 100,000: Minimal risk									
1: 100,000–1: 10,000: very low risk									

ICR_BM_ = incidence cancer risks for bone marrow; ICR_B_ = incidence cancer risks for breast; ICR_L_ = incidence cancer risks for lung; ICR_s_ = incidence cancer risks for solid cancers.

## 2 Experimental design, materials and methods

### 2.1 Data collection

Data was collected during chest radiography of young adult females (aged 17–25 year) at the x-ray unit of Radiology Departments of Obafemi Awolowo University Teaching Hospital Complex Ile-Ife, Osun State, Nigeria. The participants were students admitted into one of the Schools of the University Teaching Hospital for the year 2017. Consent was obtained from each participant before the commencement of the examination. Entrance surface dose (ESD) were determined using thermoluminescent dosimeters (TLD-100: LiF: Mg, Ti) from RadPro International GmbH, Poland. Each of the TLD chip was enclosed in labelled black polythene pack. A total of three coded chips were used to measure the entrance surface dose (ESD) during the procedure in order to obtain the mean and enhance precision. The chips were attached to an elastic tape and placed in the centre of x-rays field where the beam intercepted with the irradiated part of the patient. Patient׳s clinical information and exposure parameters were noted and recorded using self-structured form. The x-ray machine output parameters were determined using MagicMax quality control kits (IBA Dosimetry, Germany).

### 2.2 Data collection tool

The TLD chips were oven-annealed using Carbolite oven made in England. Irradiation of TLD chips for calibration (for TLD chips and Reader) was conducted at the Secondary Standard Dosimetry Laboratory (SSDL) of the National Institute of Radiation Protection and Research (NIRPR), Ibadan. TLD chips were read using Harshaw Reader (Model 3500) at the Department of Physics, Obafemi Awolowo University Ile-Ife.

### 2.3 Data analysis

The bone marrow dose, breast dose, lung dose and effective doses were evaluated from the measured entrance surface dose (ESD) using PCXMC software (version 20Rotation). Thereafter, BEIR VII model software was used to estimate the incidence cancer risk.

### 2.4 The study centre

The hospital is the only federal tertiary healthcare institution in the State with a population of about 4.7 million [21]. It provides tertiary, secondary and primary healthcare services to all the neighbouring States. The hospital serves as the teaching hospital of the Medical School of Obafemi Awolowo University Ile-Ife and has other six schools under its jurisdiction.

## Appendix A Supplementary material

Table 1 Radiological parameters of chest radiography of each patient (See Supplementary data).
