# Supplementary material for: Cancer risks from chest radiography of young adults: A pilot study at a health facility in South West Nigeria
Source: Data Brief. 2018 May 26;19:1250–6. doi: 10.1016/j.dib.2018.05.123 (PMC6140361; doi:10.1016/j.dib.2018.05.123)
Supplement: Supplementary file 1 — Supplementary material [file mmc1.doc]

**COVENANT UNIVERSITY**

**COLLEGE OF SCIENCE & TECHNOLOGY**

**DEPARTMENT OF PHYSICS**

**CANAANLAND, KM 10, IDIROKO ROAD**

**P.M.B. 1023, OTA, OGUN STATE, NIGERIA**

[**www.covenantuniversity.edu.ng**](http://www.covenantuniversity.edu.ng/)**; phy.**c**ovenantuniversity.edu.ng**

**External Memo**

**To:** Editor, DIB

**From:** Corresponding Author

**Date:** May 11, 2018

**Subject:** **Conflict of Interest**

Dear Sir,

I hereby declare that there is no conflict of interest among any of the authors.

The authors have read the final draft and have agreed that the manuscript be sent for review.

Thanks.


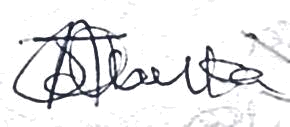


Justina A. Achuka

Department of Physics

Covenant University Ota, Nigeria

[justina.achuka@covenantuniversity.edu.ng](mailto:justina.achuka@covenantuniversity.edu.ng)

Google Scholar: Justina Achuka

Research Gate: Justina Achuka

Scopus ID: 57188717384
